# A Controlled Trial of the Knowledge Impact of Tuberculosis Information Leaflets among Staff Supporting Substance Misusers: Pilot Study

**DOI:** 10.1371/journal.pone.0020875

**Published:** 2011-06-17

**Authors:** Anjana Roy, Ibrahim Abubakar, Ann Chapman, Nick Andrews, Mike Pattinson, Marc Lipman, Laura C. Rodrigues, Jose Figueroa, Surinder Tamne, Mike Catchpole

**Affiliations:** 1 Health Protection Services, Health Protection Agency, Colindale, London, United Kingdom; 2 Department of Infection and Tropical Medicine, Royal Hallamshire Hospital, Sheffield, United Kingdom; 3 Operations Department, Crime Reduction Initiatives, Brighton, United Kingdom; 4 Royal Free Hospital NHS Trust, Respiratory Medicine, Royal Free Hospital, London, United Kingdom; 5 Epidemiology and Population Health, London School of Hygiene and Tropical Medicine, London, United Kingdom; 6 Department of Public Health, NHS City and Hackney, London, United Kingdom; McGill University, Canada

## Abstract

**Background:**

Information leaflets are widely used to increase awareness and knowledge of disease. Limited research has, to date, been undertaken to evaluate the efficacy of these information resources. This pilot study sought to determine whether information leaflets developed specifically for staff working with substance mis-users improved knowledge of tuberculosis (TB).

**Method:**

Staffs working with individuals affected by substance mis-use were recruited between Januar*y* and May 2008. All participants were subjectively allocated by their line manager either to receive the TB-specific leaflet or a control leaflet providing information on mental health. Level of knowledge of TB was assessed using questionnaires before and after the intervention and data analysed using McNemar's exact test for matched pairs.

**Results:**

The control group showed no evidence of a change in knowledge of TB, whereas the TB questionnaire group demonstrated a significant increase in knowledge including TB being curable (81% correct before to 100% correct after), length of treatment required (42% before to 73% after), need to support direct observation (18% to 62%) and persistent fever being a symptom (56% to 87%). Among key workers, who have a central role in implementing a care plan, 88% reported never receiving any TB awareness-raising intervention prior to this study, despite 11% of all respondents having TB diagnosed among their clients.

**Conclusion:**

Further randomized controlled trials are required to confirm the observed increase in short-term gain in knowledge and to investigate whether knowledge gain leads to change in health status.

## Introduction

Early suspicion and therefore prompt diagnosis of tuberculosis (TB) is an important component of TB control [1]. This is more likely to be achieved if the level of knowledge about TB among staff working with high risk groups is increased [2]. Social factors may be key features in promoting early diagnosis and TB treatment adherence [3]. These factors include response of family and community to TB and the patient. Patients encounter stigma in many settings which may result in delay of diagnosis and being non-adherent [4]. We believe that increasing knowledge among those supporting and caring for TB patients will lead to an increased understanding about TB and help overcome some of these challenges. The National Knowledge Service – TB Pilot was set up to provide information of direct relevance to people who need to be aware of, and take actions about, the treatment, prevention and management of TB in the United Kingdom [5]. The target audience are affected individuals, members of the public, healthcare professionals, and others with a duty of care.

There is a high prevalence of TB in problem drug users [6], [7]. They also often have other risk factors, such as homelessness and a history of imprisonment, associated with increased rates of TB [8]. This study aims to evaluate the effectiveness of raising awareness about TB among the group of professionals working with those affected by substance mis-use, as it has been shown that tailoring substance abuse treatment for ‘at risk' groups has the potential to control infectious diseases [9]. A recent case-control study in London showed that smear-positive disease is 2.4 times more likely to be diagnosed in crack cocaine users than in non-drug users [8]. Poor knowledge among those caring for substance mis-users may lead to mismanagement of the care of the clients and patients, adversely affecting outcome. Previous reports also suggest that increasing TB awareness may raise short term knowledge [5]. However, there is limited data to demonstrate any effect on transmission [5], [10].

The National Knowledge Service for TB developed information leaflets, distilled from current evidence-based guidance and expert opinion on TB, for staff working with individuals affected by substance mis-use. This leaflet was piloted for the first time in this study. Here, we report a pilot study on how to evaluate the effectiveness of targeted information delivery through context-specific TB leaflets to improve knowledge of TB among key workers who support recently released offenders with a history of substance mis-use. Key workers in this study are the professionals providing specialist services to those affected by substance mis-use. TB knowledge gain among key workers was compared to a control group who received a mental health leaflet.

## Methods

Camden & Islington Community Local Research Ethics Committee, London confirmed that the study was an evaluation and did not require full ethical review.

### Study Design

Multi-centre pilot controlled trial

### Participants

Key workers were all staff working for the national charity ‘Crime Reduction Initiatives' (CRI), involved with the resettlement and rehabilitation of offenders and those with substance mis-use needs who were approached for consent to participate. Eligible participants included all staff (n = 150) working with offenders following release from prison and those affected by substance mis-use. Study recruitment took place between January and May 2008. Respondents (key workers) included care workers, social workers, project workers, nurses and mental health workers.

### Procedure

A multi-centre pilot controlled trial was conducted among eligible staff. The study covered three areas of London (Ealing, Hounslow, and Camden), Brighton, Eastbourne, Stockton, Bognor and Regis. Participants were assigned to receive either the ‘Substance Mis-use and TB' leaflet or ‘Substance Mis-use and Mental Health' leaflet (Leaflets S1 and S2). Staff of CRI is primarily involved in the rehabilitation of offenders by offering assessment, information and advice, treatment and referral, on areas including social welfare, health, housing, education, training and employment. The materials were developed by representatives of the Health Protection Agency, National Treatment Agency, Adfam (a charity) and National Health Service (NHS) clinicians. The representatives were from different professional backgrounds, with expertise and experience in the diagnosis, control and prevention of TB and those involved in the management of clients affected by substance mis-use.

The managers of CRI allocated half their staff to the intervention (TB) leaflets and the other half to the control leaflets (mental health). The allocation was neither randomized nor blinded rather the manager simply divided an unordered list of eligible staff in two. The intervention was an information leaflet, entitled ‘Substance Mis-use and TB: Guidance for key workers', which provided information on how TB is transmitted, the likelihood of developing the disease, TB symptoms and management, along with complications of alcohol/drug misuse. The leaflet also included specific information on how the key worker could support the TB treatment of individuals in their care, and whether staff members themselves were at risk. Participants allocated to the control group received a mental health leaflet entitled “Mental Health and Substance Mis-use” (which contained no information on TB).

**Figure 1:**
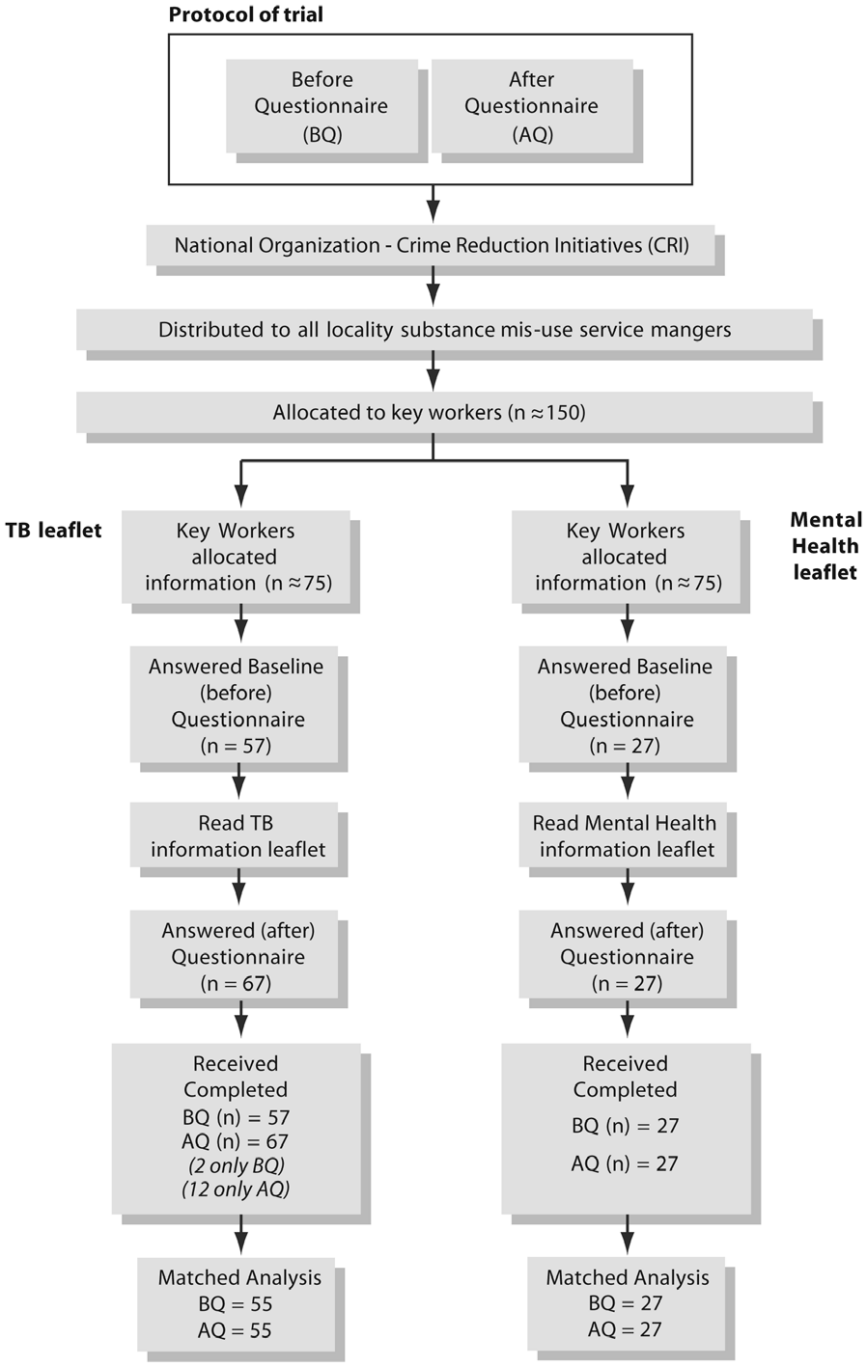
Protocol of Trial.

Regional managers of the organisation employing the participating staff were requested to allocate an equal number of TB and mental health leaflets to key workers in their group. Staff members were informed that their organisation had agreed to participate in evaluating TB information resources. They were informed that the study was a comparison study where some of the participants would receive a TB leaflet and others a mental health leaflet. Both groups were then tested on their knowledge of TB both before and after reading the leaflet. The members received the study questionnaires by e-mail (Questionnaire S1 and S2) along with the intervention or control leaflet. They were required to fill out the ‘before' questionnaire, read the assigned leaflet, and then complete the ‘after' questionnaire. Both sets of questionnaires were identical aside from the fact that the first questionnaire had some additional questions regarding the respondent's background and previous knowledge of TB. *A priori* the team agreed to answers which are acceptable and unacceptable (right or wrong) and to use qualitative assessments which were rated by respondents on a 5-point Likert scale for self assessment of TB awareness [11], [12]. Knowledge gain was measured through before and after questionnaires, which assessed the number of wrong answers that were corrected after reading the leaflet. The pre-specified outcome of the study was a change in the level of knowledge of the target users following the distribution of information leaflets (Figure 1).

The questions were based on information provided in the TB leaflet. There were 12 multiple choice questions in the ‘before’ questionnaire comprising five on the respondent's background and prior experience of TB, and seven on the clinical aspects of TB; the ‘after’ questionnaire contained the same seven clinical questions on TB issues. The questionnaires provided the workers with a list of possible answers. Some of the multiple choice answers are not related to TB. We estimated that the entire process of completing the ‘before’ and ‘after’ questionnaires, as well as reading the leaflet, would take the participants an average of 20–30 minutes.

### Statistical analysis

Scoring of responses before and after reading the TB leaflet or control leaflet was carried out, with one point given for a correct answer and no points for a wrong answer. Statistical analyses were performed using Stata version 9.2. Percentages were calculated. McNemar's exact test for matched pairs was used to test whether there was a significant change in the percentage giving a correct answer before and after each intervention. Two-tailed chi-squared test was calculated, a p value of <0.05 was deemed as being statistically significant. The difference in percentage was also calculated with McNemar's 95% confidence intervals. Comparison of pre-existing knowledge between groups was performed using Fisher's exact test and p values reported.

## Results

The questionnaires were circulated to all 150 eligible key workers: 96 responses (64%) were received. Although the same numbers of staff were allocated to the intervention and control groups, 55 individuals in the intervention and 27 in the control returned both the ‘before’ and ‘after’ questionnaires. Fourteen participants returned only 1 questionnaire (2 before and 12 after). As we conducted a matched analysis, these questionnaires were excluded from analysis (Figure 1).

### Staff background

The workers participating in this study were from across the UK. Seventy per cent (57/82) of respondents answered the background section of the questionnaire. The majority of respondents (54%; 31/57) were drug intervention programme workers, 12% (7/57) were substance mis-use workers, while case workers, project workers, and criminal justice intervention group workers each constituted 5% of respondents (3/57). Two per cent of respondents each were social workers, nurses, and staff involved in the protection of sex workers (2/57). In addition, a harm minimization worker, a structured programme worker, a member of the arrest referral team, and a mental and community health worker also answered the questionnaire.

### Staff awareness of TB before reading the resources

Staff were asked to self-assess their knowledge of TB using one of five options: very good, good, average, below average and poor. Levels of self-reported knowledge of TB were similar among the intervention and control group with 66% and 62% respectively reporting ‘below average' knowledge. Fifteen percent of respondents in both groups (8/55 in the intervention) and ( 4/27 in the control group) reported that they had never read any TB leaflets or attended any TB awareness-raising intervention prior to this study. Thirteen per cent (7/55) of staff in the intervention and 7% (2/27) of staff in the control group reported having had a client diagnosed with active TB in their care at some point - suggesting a high incidence of TB in this client group.

### Knowledge Gain

#### General knowledge about TB

**Figure 2:**
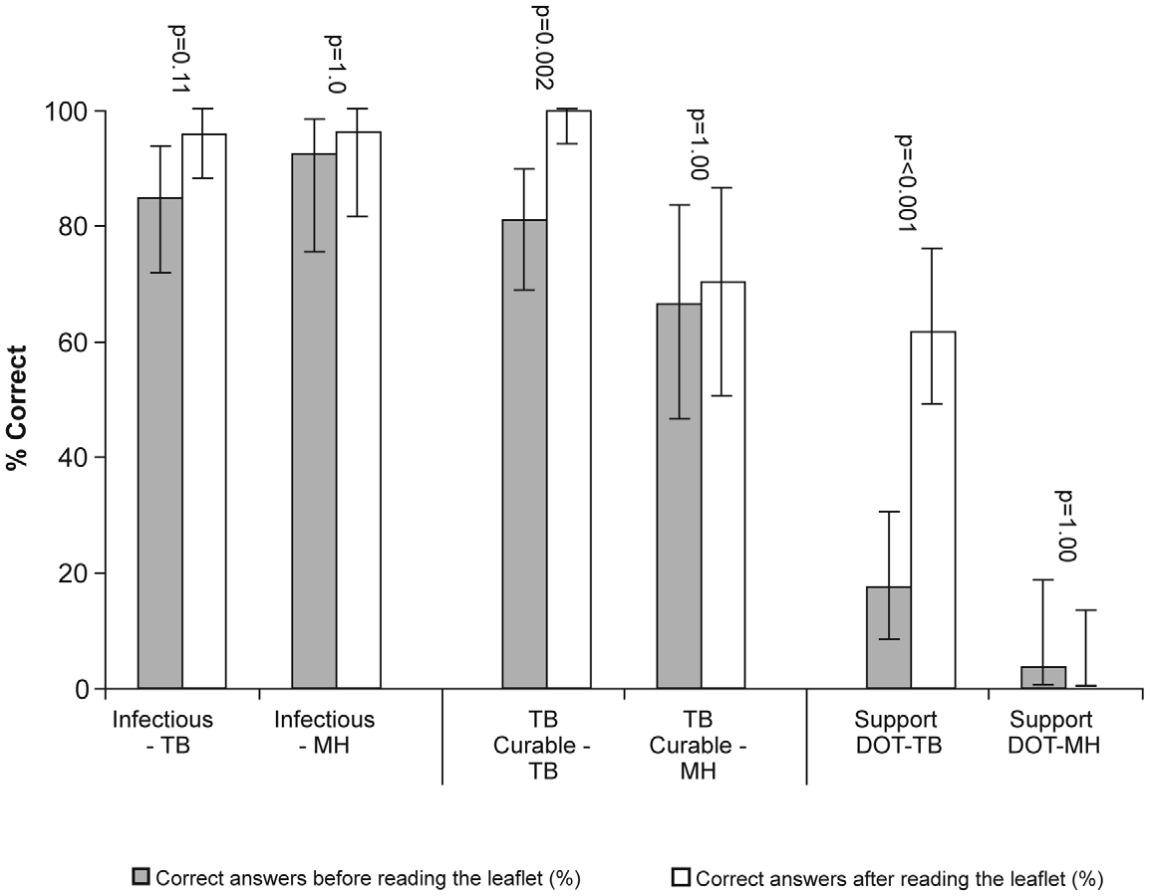
Examples of gain in knowledge before and after reading the TB leaflet and the Mental Health leaflet.

Pre-existing knowledge of TB was high in both groups for some areas of knowledge, such as pulmonary TB being the infectious form (Figure 2). Over 85% of all health and social care professionals involved in the study were aware of where to direct their clients before the intervention, irrespective of whether they had read the TB or Mental Health leaflets (see Table 1). Comparing the pre-existing knowledge between groups showed no evidence of a significant difference for general knowledge, symptoms or treatment issues.

**Table 1:** Change in knowledge before and after reading the TB and control leaflet

	Tuberculosis leaflet (N = 57)			Mental Health leaflet (N = 27)		
	%correct before	%correct after	%change in correct (95%CI), p value*	%correct before	%correct after	%change in correct (95%CI), p value*
**Knowledge of symptoms of TB**						
Itchiness	100(57/57)	98(56/57)	−2 (−7 to 3), 1.00	81(22/27)	81(22/27)	0 (−14 to 14), 1.00
Persistent fever	56(32/57)	87(50/57)	31 (15 to 47), 0.0002	52(14/27)	52(14/27)	0 (−18 to 18), 1.00
Night sweating	53(30/57)	89(51/57)	36 (21 to 52), <0.0001	44(12/27)	48(13/27)	4 (−7 to 14), 1.00
Unusual tiredness	65(37/57)	82(47/57)	16 (−1 to 34), 0.08	52(14/27)	44(12/27)	−7(−21 to 6), 0.50
Stomach cramping	96(55/57)	93(53/57)	−4 (−12 to 5), 0.62	85(23/27)	90(24/27)	4 (−12 to 20), 1.00
Weight loss	58(33/57)	93(53/57)	34 (17to 52), 0.0002	67(18/27)	63(17/27)	−3 (−14 to 7.1), 1.00
Cough for long	73(42/57)	82(47/57)	9 (−7 to 25), 0.33	74(20/27)	74(20/27)	0 (−14 to 14), 1.00
Coughing up blood	78(44/57)	98(56/57)	20 (8 to 32), 0.001	85(23/27)	89(24/27)	4 (−7 to 14), 1.00
Bone fracture	96(54/57)	96(55/57)	0 (− 9 to 9), 1.00	96(26/27)	96(26/27)	0 (−3 to 3), 1.00
**Understanding the need for referral**						
Do nothing	92(52/57)	98(56/57)	5 (−2 to 13), 0.25	96(26/27)	100(27/27)	4 (−7 to 14), 1.00
Specialist services / GP	89(51/57)	87(50/57)	−2 (−11 to 8), 1.00	89(24/27)	89(24/27)	0 (−18 to 18), 1.00
Transfer to another hostel	100(57/57)	98(56/57)	−2 (−7 to 3), 1.00	93(25/27)	100(27/27)	7 (−6 to 21), 0.50
Contact Public Health Lab	60(34/57)	74(42/57)	14 (−2 to 31), 0.10	74(20/27)	74(20/27)	0 (−14 to 14), 1.00
**Knowledge of treatment issues**						
TB curable	81(46/57)	100(57/57)	18 (6 to 30), 0.002	67(18/27)	70(19/27)	4 (−12 to 20), 1.00
Client responsible medication	27(15/57)	44(25/57)	16 (−1 to 33), 0.08	22(6/27)	26(6/27)	4 (−12 to 20), 1.00
Length of treatment	42(24/57)	73(42/57)	31 (14 to 47), 0.005	33(9/27)	41(11/27)	7 (−6 to 21), 0.50
Monitoring progress	42(24/57)	47(27/57)	5 (−11 to 22), 0.63	44(12/27)	44(12/27)	0 (−4 to 4), 1.00
Interaction medications	85(49/57)	93(53/57)	7 (−3 to 18), 0.22	89(24/27)	85(23/27)	−3 (−20 to 12), 1.00
Medication Charges	100(57/57)	98(56/57)	−2 (−7 to 3), 1.00	96(26/27)	100(27/27)	4 (−7 to 14), 1.00
**Understanding the need to provide support and general awareness**						
Infectious form of TB	85(49/57)	96(55/57)	11 (−2 to 24), 0.11	93(25/27)	96(26/27)	4(−7 to 14), 1.00
Meaning of DOT	9(5/57)	36(21/57)	27 (12 to 43), 0.0007	0(0/27)	7(2/27)	7 (− 6 to 21), 1.00
Support DOT	18(10/57)	62(35/57)	44 (29 to 59), <0.0001	4(1/27)	0(0/27)	−4 (−14 to 7), 1.00
Client's background	64(36/57)	71(40/57)	7 (−8 to 22), 0.42	81(22/27)	89(24/27)	7 (−6 to 21), 0.50
Colleagues − risk of TB	54(31/57)	69(39/57)	14 (−3 to 32), 0.11	56(15/27)	63(17/27)	7 (−14 to 29), 0.68
Sharing items	73(42/57)	87(50/57)	14 (2 to 27), 0.02	70(19/27)	70(19/27)	0 (−18 to 18), 1.00

*McNemar's p value, CI – confidence interval, TB – Tuberculosis, DOT – directly observed therapy, GP – general practitioner.

In the group allocated the TB leaflet there were significant increases in the proportion of correct answers in the post-leaflet questionnaire for a number of questions relating to general knowledge of TB, including its ability to be cured which increased from 81% (46/57) to 100% (57/57) in the intervention group and from 68% (18/27) to 70% (19/27) in the control group (p = 0.002). Knowledge of the minimum length of treatment required increased from 42% (24/57) to 73% (42/57) in the intervention group and 33% (9/27) to 41% (11/27) in the control group after reading the leaflet (p = 0.005) (Table 1). There was no knowledge gain for any question in the control group.

#### Symptoms of TB

There was a significant increase in the number of correct answers for several questions on TB symptoms from those who read the TB leaflets, compared to those given the control leaflet. These significant increases were for: persistent fever (intervention group: before 56% (32/57), after 87% (50/57) p = 0.0002); control both before and after 52% (14/27) p = 1.0); night sweats - intervention group: before 53% (30/57) after 89% (51/57) p<0.001; control before 44% (12/27) after 48% (13/27) p = 1.0); weight loss - intervention group: before 58% (33/57) after 93% (53/57) p = 0.0002; control before 67% (18/27) after 63% (17/27)p = 1.0); and coughing up blood – intervention group: before 78% (44/57) after 98% (56/57)p = 0.001; control before 85% (23/27) after 89% (24/27) p = 1.0). Key workers in both groups were aware that itchiness, unexpected bone fracture and stomach cramps are not symptoms of TB (Table 1).

For the symptoms of unusual tiredness and prolonged coughing, there was a slight increase among those who corrected their answer after reading the TB leaflet, but this was not significant (intervention group: before 65% (37/57) after 82% (47/57) p = 0.08 and before 73% (42/57) after 82% (47/57) 0.33; control before 52% (14/27) after 44% (12/27) p = 0.5 and both before and after 74% (20/27) p = 1.0 respectively).

#### Treatment Issues and Supporting Clients

Among those who read the TB leaflet there was a significant increase in the proportion of staff who knew that they, or a member of their team, may be asked to watch clients take their medication, as well as the proportion of key workers who understood that may be required to support direct observation of treatment (Table 1). In contrast, there was no change in knowledge about either of these factors among those who read the mental health leaflets. The group that read the TB leaflet also showed a significant (p = 0.02) increase in knowledge regarding risk of infection through sharing household items such as bed linen, crockery and linen, with no change in knowledge in the control group.

## Discussion

This pilot study assessed knowledge of TB in a group of individuals who work with substance mis-users and paves the way for a larger study taking on board the limitations of this study. The target audience for the resource evaluated in this study was fairly diverse, though often worked in settings that would be classified as healthcare services. Substance misuse is a risk factor for TB in the UK, and our finding that 11% of respondents had looked after a patient with TB demonstrates the high prevalence of TB in this group. The majority of respondents self-reported their knowledge of TB as below average - highlighting the importance of raising TB awareness, as outlined in the Chief Medical Officer's Action Plan published in 2004 [13]. This was also emphasized two years later within the National Institute for Health and Clinical Excellence (NICE) TB guidelines [14]. Initiatives such as the National Knowledge Service TB Project have been set up to raise awareness of TB in high-risk individuals and the professionals looking after them. There is some evidence from previous studies that providing information in a context-specific format can increase knowledge of TB [5]. Limited research has, to date, been undertaken to evaluate the efficacy of information resources [15]. Here we have demonstrated that leaflets may deliver short-term knowledge gain with regard to awareness of symptoms of TB, an understanding of risks of infection, as well as measures required to support the treatment of those affected. These findings would, however need to be confirmed by a larger trial.

There is an increased drive to use awareness as a measure for TB control and to improve the general lack of knowledge and several misconceptions about TB from various countries [16]–[19].

The overall results of the ‘before’ questionnaire suggest variable levels of knowledge concerning the symptoms of TB: only about 50% knew that TB causes fever (Figure 2), sweats and weight loss, while almost all respondents indicated that itchiness, stomach cramps and fractures were not associated with TB. Around three quarters of respondents said that TB was curable (Figure 2), and over 80% were aware of the interactions between TB medications and illicit drugs - an interesting finding considering that other knowledge on TB treatment was more limited.

Very few respondents to the ‘before’ questionnaire had knowledge of directly observed therapy (Figure 2), or their own potential role in supporting TB therapy. This is important as TB patients who are substance mis-users are more likely than non-drug users to default from treatment, to remain infectious for prolonged periods after diagnosis and to acquire drug resistance [6]. The need to conform to specific diagnostic tests before supplying opiate substitutes due to potential for overdose caused by non-adherence makes DOT especially valuable to this group. Directly observed therapy is only recommended by NICE in this group after a risk assessment [20], other studies suggest that these patients may benefit from implementing DOT. The key workers have a central role in co-ordinating a care plan and building a therapeutic alliance with the service user.^4^ Therefore, improving knowledge about TB in this professional group is likely to improve outcome for patients in terms of early diagnosis and treatment completion, as well as contribute to prevention and control of the disease nationally.

There are many published examples of targeted provision of information to raise awareness. In diabetes care and clinical trial awareness-raising programmes, the provision of materials in several formats seeking to meet the needs of different audiences is well documented [21], [22]. For HIV/AIDS, recent studies have shown that both professional and non-professional care-givers make use of internet information resources to support patients [23], and that HIV-positive individuals themselves also use the internet to search for HIV-specific information and support [24]. In some cases, use of such information resources has been demonstrated to either raise awareness and knowledge levels [5], [14] or alter behaviour or other outcomes [25], [26]. However, although poorly researched, there is no consistent evidence that public education campaigns lead to sustained change in behaviour or improved health outcomes [27]. A recent study screening migrants for TB in Sweden recommended that appropriate and accessible educational programmes are needed to address misconceptions and to change attitudes, and thus bring about TB control [28].

### Study limitations and implications for future studies

As this is a pilot study, a number of limitations are acknowledged. We did not assess either long-term knowledge retention or changes in behaviour in our study. We recognise that further measures to provide ongoing guidance on how staff can help and support their clients may be required to sustain the knowledge gain demonstrated here. Other limitations of this study are: (i) participants read the leaflet knowing that they had to answer questions later, and as such may have been more likely to focus their attention on what information they needed to get from the reading than would have been the case in other circumstances; (ii) the allocation of the intervention or control resources was not formally randomized; (iii) as the questionnaires and respective leaflets were sent simultaneously, there is no guarantee that such knowledge would affect practice. Ideally, re-testing should have been carried out; (iv) the study had to rely on respondents' honesty and this may have encouraged those with the Mental health leaflet (control leaflet) to be consistent with their replies, and those with TB leaflet to change to the correct response; and (v) the response rate in the control group was approximately half that of the intervention group. This may have been due to a lack of confidence and unwillingness on the part of the control group to return questionnaires specifically testing knowledge of TB, since they had not been provided with information on TB. To minimise the potential for bias arising from the non random allocation of participants, we checked whether the respondents in the control and intervention groups differed by profession. The small number of health care workers and the comparable proportion of responses in the two groups before and after the intervention suggest that there is a low likelihood of this bias.

Due to these limitations our results do not provide definite evidence for policy change but point to the potential benefits that require confirmation. Despite these potential limitations, we believe that our pilot study provides useful preliminary data and identifies key issues that need to be considered in a future randomised controlled trial. Further studies should assess a change in health status as the primary outcome measure. It is likely that a large sample size will be required to demonstrate the probable modest beneficial effect of awareness rising on tuberculosis transmission and/or treatment completion. Other intermediate outcomes, such as behaviour change, should be considered. The study should ensure an adequate sample size to detect a modest effect of public health importance as suggested by the percentage change in knowledge from this study. Further considerations include that the intervention should be delivered in such a way that allows adequate double blinding and that short term and long term knowledge gain are assessed as secondary outcomes.

## Conclusion

Further randomized controlled trials are required to confirm the observed increase in short-term gain in knowledge and to investigate whether knowledge gain leads to change in health status.

## Supporting Information

Leaflet S1 National Knowledge Service - TB - Health Protection Agency, National treatment Agency and Adfam: Substance misuse and TB: Guidance for key workers (care workers, social workers, project workers and health professionals). 2007. Health Protection Agency.
(PDF)

Leaflet S2 Adfam and rethink. Mental health and substance misuse : information for families, firends and carers. 2004.
(PDF)

Questionnaire S1 National Knowledge Service TB : PART 1 Evaluation of information resource :*TB* & *substance misuse: Guidance for substance misuse – key workers*. 2008. Health Protection Agency.
(PDF)

Questionnaire S2 National Knowledge Service TB : PART 2 Evaluation of information resource : *TB* & *substance misuse: Guidance for substance misuse - key workers*. 2008. Health Protection Agency.
(PDF)
