# Supplementary material for: A Controlled Trial of the Knowledge Impact of Tuberculosis Information Leaflets among Staff Supporting Substance Misusers: Pilot Study
Source: PLoS One. 2011 Jun 17;6(6):e20875. doi: 10.1371/journal.pone.0020875 (PMC3117836; doi:10.1371/journal.pone.0020875)
Supplement: Leaflet S1 — National Knowledge Service - TB - Health Protection Agency, National treatment Agency and Adfam: Substance misuse and TB: Guidance for key workers (care workers, social workers, project workers and health professionals). 2007. Health Protection Agency. (PDF) [file pone.0020875.s001.pdf]

NHS
National Treatment Agency for Substance Misuse

Adfam
Families drugs and alcohol

Health Protection Agency

Substance misuse and TB:

Guidance for key workers

(care workers, social workers, project workers and health professionals)

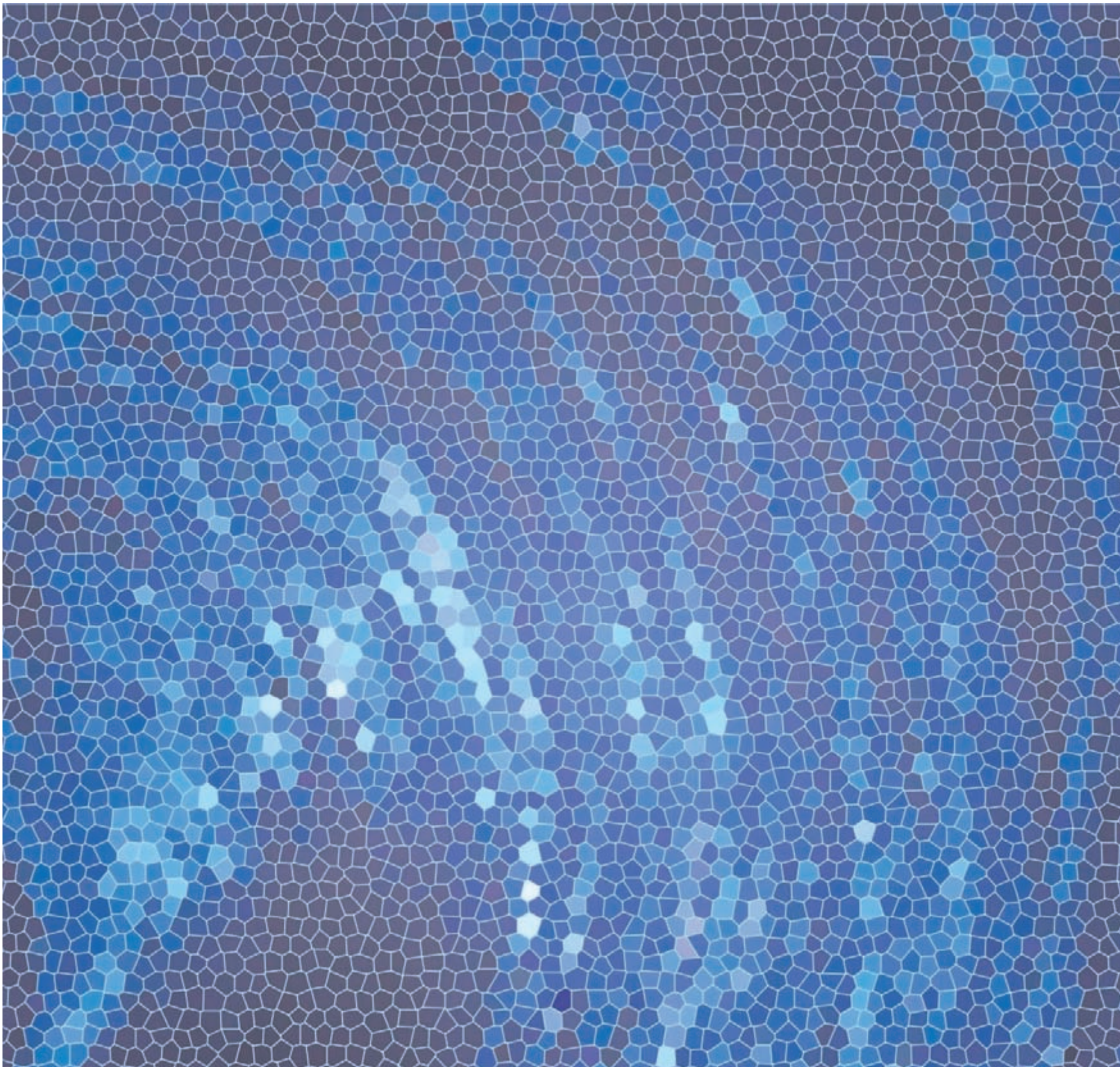

What is TB?

Tuberculosis (TB) is a bacterial airborne infectious disease that spreads between people following prolonged contact. TB rates in the UK are on the increase, and individuals with a substance misuse problem are around 10 times more likely to get the disease relative to the general population. Not all forms of TB are infectious: only lung TB may be (but not always) passed on from one individual to another.

Spotting symptoms & referrals

Early detection of TB makes it easier to treat and reduces onward transmission. It is important that you are aware of the signs and symptoms of TB so that you can increase awareness among your clients.

If you think a client may have TB you should refer them to appropriate health providers.

Think TB! - *Spotting symptoms*

Look out for a cough which persists for more than three weeks, and two or more of the following symptoms, your client may have TB. They should ask to see a nurse or doctor urgently.

- Persistent fever
- Heavy sweating at night
- Loss of appetite
- Unexplained weight loss
- General & unusual sense of tiredness and being unwell
- Coughing up blood
- Recent contact with someone who has TB.

Remember - Just because someone has had the vaccination for TB (BCG) does not mean that they cannot get TB!

Providing assistance

Vulnerable clients/patients may need support in accessing health services and should, where possible, be accompanied by a member of staff who can advocate for them.

Referral pathway for tuberculosis among alcohol / drug related users

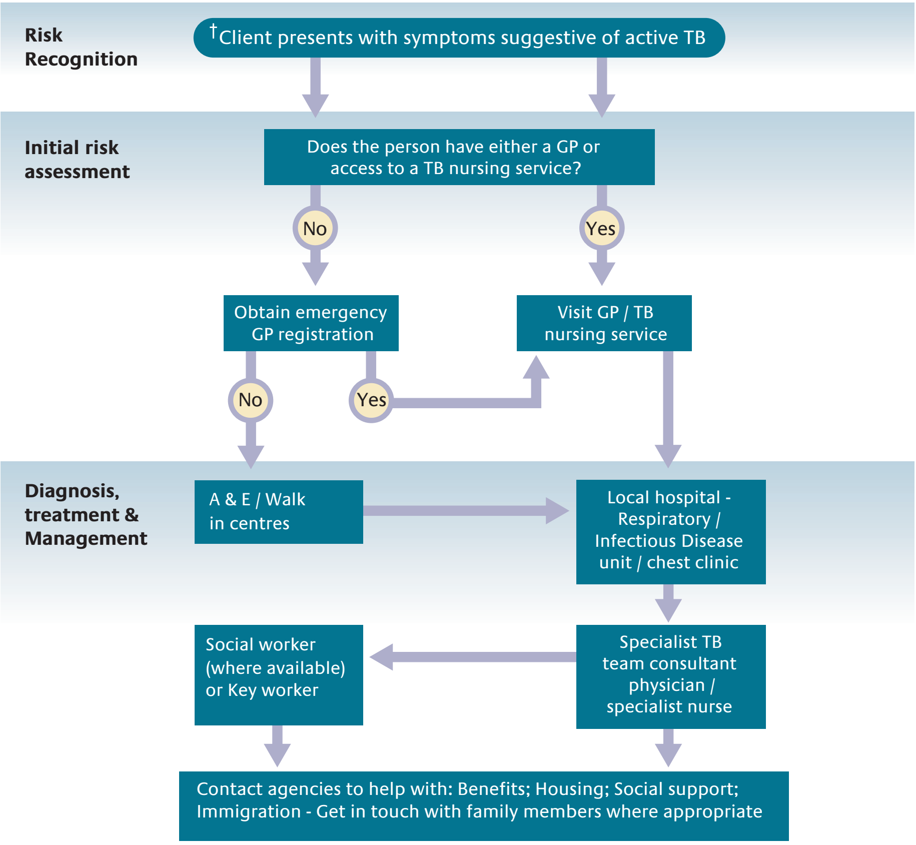

†Anyone who is coughing up blood requires urgent assessment

Where should I direct clients for help?

Please see referral pathway

You should ask them / take them to see their GP or local TB nursing service if they have one. If not, you should help or advise them to obtain emergency GP registration, take them to a walk-in centre, or talk to health care professionals that you might work with e.g. drug / alcohol workers or doctors who should know what to do. If none of these options is possible then they should be directed to the local hospital emergency department. Support the client by accompanying them to their appointments where possible.

What is my role?

- **Be aware** of the symptoms of TB, and if you think that one of your clients is showing these symptoms, encourage them to seek medical assistance.
- **Support** diagnosis, treatment and aftercare of the individual. For those on treatment :
  - motivate them to complete the full course
  - support them to keep their follow up appointments
- **Encourage** clients to express thoughts, feelings, or information easily. Ask about family circumstances with a view to offering support and involve family where possible.
- **Provide** appropriate information about the patient to medical staff.

Can TB be cured?

Yes – TB is completely curable PROVIDED they take their tablets regularly for the full length of treatment, which is at least 6 months.

How will TB be diagnosed?

- A doctor or nurse will arrange for your client to have some tests. This may involve testing their phlegm and having a chest x-ray.
- Your client may be referred to a specialist TB or chest clinic for these investigations, or they may be arranged by a GP.

Treatment

- If a client is diagnosed with infectious TB (not all TB cases are infectious), they may be admitted to hospital and treated in a single room until they are non-infectious (usually 2 weeks). He/she will then be discharged home to continue with their course of treatment and regular clinic follow-up. If they are not infectious they may start their treatment in a TB / chest clinic or at home.

- If they stop taking the tablets, the disease could come back and be more difficult to treat because of drug resistance. Patients may continue to be infectious for a longer time and pass the disease on to family and friends.
- Medication for your client will be supplied free of charge from a TB / chest clinic.

How can I support my client to take regular medication?

- Getting clients to take a full course of TB treatment is the most challenging obstacle to TB control. You are well placed to support clients with TB through the care period and ensure that they take their medication as prescribed.

Directly Observed Therapy (DOT)

Directly Observed Therapy is watching the patient take his/her medication to ensure that this is taken in the right combination and for the correct length of time. DOT is recommended for all patients with a poor record of taking medication; or where there are concerns that they may not be able to take it as prescribed by the TB team.

Directly Observed Therapy should be organised by the person's care manager (usually a member of the TB healthcare team) but can be carried out by anyone in regular contact with the client. Medication can be provided for each client in pre-prepared blister packs or dosset boxes. Staff would not necessarily be administering medication but supporting the client in completing treatment and reporting any missed doses to the person's care manager.

What happens if the client continues drinking alcohol during treatment for TB?

- There will be a high risk of liver damage. Regular blood tests will need to be done to ensure that the liver is functioning properly.

What happens if the client continues to take substitute medication during treatment for TB?

- It is safe to take the two sets of treatment together, as long as the treatment is carefully monitored by a healthcare team. Substitute drugs, such as methadone, and TB medication affect each other. When your client starts treatment for TB they may require an increased substitute drug dose. The dose will then need to be reduced at the end of TB treatment or the client may overdose and could die. The same applies if your client is taking illicit drugs. It is very important to be aware of any medication he/she is taking.
- Close communication between TB services, prescribing agencies and clients is VITAL for successful recovery.

What happens if the client is co-infected with Hepatitis B or C, or HIV?

- One of the commonest side-effects of TB drugs is liver damage, and this is more likely with Hepatitis co-infection. The client will therefore need regular blood tests to monitor their liver.
- Anti-retroviral therapy (HIV medication) taken together with TB treatment may have side-effects and interactions requiring careful monitoring by the healthcare provider. Your client's treatment will be monitored more closely; please make sure that they keep their appointments.

What kind of information should I have?

You will need to provide only as much information about your client as is normally required.

The medical team may need to know the following:

- Contact details for the client.
- Treatment / drug regime they are on, whether prescribed or non-prescribed.
- Any other medical issues.

It will be very useful if you could maintain contact with your client's healthcare and social workers. This will help to maintain continuity of treatment and care. All information provided to the health services will be treated in strictest medical confidence.

Will arrangements have to be made to decontaminate our premises?

Decontamination is not necessary if a client has been diagnosed with TB. Hence there is no need for fumigation of rooms or separate kitchen facilities/crockery/ bed-linen etc.

What are the implications of a case of TB for me or my staff?

TB is usually only infectious after prolonged close contact. Awareness of symptoms and treatment regimens are crucial to ensure that TB cases are detected early and spread is reduced. If there are concerns your staff can be checked by members of your local TB services.

- All members of staff should be aware of symptoms, treatment and what to do if they suspect that they, a client or other members of staff have TB. TB awareness should be a part of induction processes as well as follow-up health awareness training.

Need to know more?

Further information and contacts are available on:

HPA website: www.hpa.org.uk/infections/topics_az/tb/menu.htm

National Knowledge Service - TB Pilot: www.hpa.org.uk/tbknowledge/default.htm

National Treatment Agency: www.nta.nhs.uk

Adfam: www.adfam.org.uk

You can also call NHS Direct on: 0845 4647 or go to **www.nhsdirect.nhs.uk**

Your local Health Protection Agency

....

....

....

....

....

To find your local health protection unit go to:
www.hpa.org.uk/lars_homepage.htm

Your local Primary Care Trust (PCT)

....

....

....

....

....

To find your local PCT go to:
www.nhs.uk/ServiceDirectories/Pages/PrimaryCareTrustListing.aspx

Your local TB / Respiratory nurse can be contacted at:

Health Protection Agency
Centre for Infections
61 Colindale Avenue
London
NW9 5EQ

Tel 020 8200 4400
www.hpa.org.uk

October 2007
© Health Protection Agency
