# Supplementary material for: A Controlled Trial of the Knowledge Impact of Tuberculosis Information Leaflets among Staff Supporting Substance Misusers: Pilot Study
Source: PLoS One. 2011 Jun 17;6(6):e20875. doi: 10.1371/journal.pone.0020875 (PMC3117836; doi:10.1371/journal.pone.0020875)
Supplement: Leaflet S2 — Adfam and rethink. Mental health and substance misuse : information for families, firends and carers. 2004. (PDF) [file pone.0020875.s002.pdf]

Where can I get help?

For medical advice or access to services, contact your GP, psychiatrist, or community psychiatric nurse. Rethink can offer help and support, and Adfam can offer access to support for families and carers.

Organisations that others have found helpful in giving support and advice on this type of problem include:

Adfam – A national organisation that aims to raise awareness of, and take action to alleviate, family problems associated with drugs and alcohol.

Address: Waterbridge House, 32-36 Loman Street, London SE1 0EH. Tel: 020 7928 8898. www.adfam.org.uk

Alcoholics Anonymous (AA) – A national organisation with many local groups. AA brings people together to share their experiences and provide mutual support.

Address: General Services Office, PO Box 1, Stonebow House, Stonebow, York YO1 2NJ
Helpline: 0845 7697 555. www.alcoholics-anonymous.org.uk

Al-Anon Family Groups – Offers understanding and support to the families of problem drinkers.

Address: 61 Great Dover Street, London SE1 4YF
Tel: 020 7403 0888 (Open 10am – 4pm)
Out of hours numbers: 020 7407 8180 or 020 7407 7878

Alcohol Concern – Comprehensive information on alcohol and on local service providers.

Address: Waterbridge House, 32-36 Loman Street, London SE1 OEE. Tel: 020 7928 7377
www.alcoholconcern.org.uk

Drinkline – A free and confidential helpline for anyone concerned about their own or someone else's drinking.

Tel: 0800 917 8282 (Lines open 11am – 11pm Mon– Thurs and 24 hours a day on Fri, Sat and Sun)

DrugScope – UK independent centre of expertise on drugs with an extensive library of drug information. Also promotes effective responses to drug taking, undertakes research at local, national and international levels, advises on policy-making, encourages informed debate and speaks for member organisations working on the ground.

Address: 32-36 Loman Street, London SE1 OEE
Tel: 020 7928 1211. www.drugscope.org.uk

FRANK – Campaign from the Department of Health and the Home Office, supported by the DfES. Information and advice on drugs to anyone concerned about drugs and solvent/ volatile substance misuse, including drug misusers, their families, friends and carers. Information and advice is available in several languages.

Confidential, daily 24 hour service
Tel: 0800 77 66 00 www.talktofrank.com

NHS Smoking Helpline
Tel: 0800 169 0169 (Lines open 7 days a week 7am – 11pm. Specialist advisors available from 10am – 11pm)
www.givingupsmoking.co.uk or www.ash.org.uk

Rethink severe mental illness – has more than 30 years experience of helping people with severe mental illness and their families recover a meaningful life. As well as running over 400 mental health services, Rethink has a network of more than 120 support groups across the country.

Address: 28 Castle Street, Kingston upon Thames, Surrey KT1 1SS Tel: 0845 456 0455
Fax: 020 8547 3862 E-mail: info@rethink.org
www.rethink.org

Revolving Doors Agency – Aims to improve the quality of life of people with mental health problems in contact with the criminal justice system.

Address: The Turnmills, 63 Clerkenwell Road, London EC1N 5NP. Tel: 020 7253 4038
www.revolving-doors.co.uk

Turning Point – Turning Point is the UK's leading social care organisation providing services for people with complex needs across a range of health and disability issues. It is the largest provider of substance misuse treatment services and a major provider of mental health and learning disability services. Last year, Turning Point had contact with almost 100,000 people through services in 200 locations in England and Wales. Turning Point provides services for people with concurrent mental health and substance misuse problems.

Address: New Loom House, 101 Back Church Lane, London E1 1LU. Tel: 020 7712 2300. www.turning-point.co.uk

Generic carer support groups

Carers UK – Offers support and information to carers.

Address: Ruth Pitter House, 20-25 Glasshouse Yard, London EC1A 4JT
Tel (office): 020 7490 8818 Tel (Helpline): 0808 808 7777
(open Mon–Fri, 10a –12pm and 2pm–4pm)
E-mail: info@ukcarers.org www.carersonline.org

Equip Carer Support – Provides lists of national and regional carer support organisations. It also provides carer information on specific medical topics, information for parents and families and on children's health, disability support and social care services www.equip.nhs.uk/groups/carer.html

www.careuk.net – Provides a selection of useful links for carers with detailed descriptions.

Parentlineplus – Provides support and information to anyone who parents a child. Tel: 0800 8000 2222
www.parentlineplus.org.uk.

This project has been made possible by the support and funding of the Home Office.

Rethink acknowledges with grateful thanks the help of Gabrielle Ayerst and Vivienne Evans in preparing this leaflet.

Copyright 2004 © Rethink and Adfam

All rights reserved. No part of this leaflet may be reproduced or transmitted in any form or by any means, electronic or mechanical, including photocopying, recording, or by any information storage and retrieval system without the permission, in writing, of Rethink Severe Mental Illness.

Rethink is the operating name of the National Schizophrenia Fellowship.

Rethink registered charity no: 271028
Adfam registered charity no: 1067428

Adfam Families, drugs and alcohol
rethink severe mental illness

Understanding Dual Diagnosis

Mental health and substance misuse

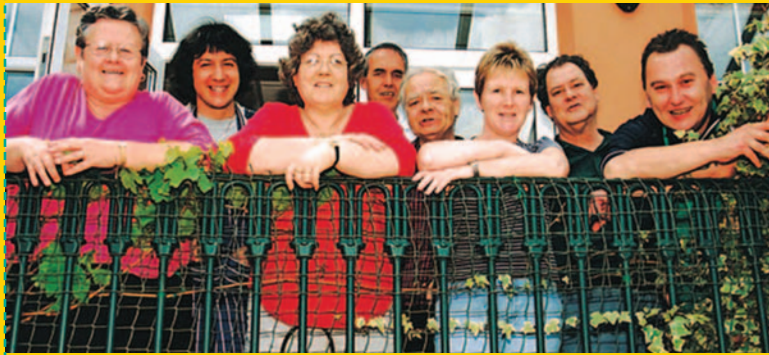

Information for families, friends and carers

Adfam Families, drugs and alcohol
rethink severe mental illness

Introduction

People who have problems with both mental health and misuse of drugs or alcohol often need a lot of support from families and friends. If you are supporting a person with "dual diagnosis" this leaflet is intended to offer advice and information. It clarifies what the term dual diagnosis means, outlines the care on offer and suggests ways in which you can help. Most importantly, it provides information about sources of advice and support – both for the individual and for you as a carer.

What is dual diagnosis?

The term describes people who have severe mental health and drug or alcohol problems. The mental health problems may include schizophrenia, depression or bipolar disorder (manic depression) or personality disorders.

Why do people have both problems?

There are many reasons why people with mental health problems may turn to drugs or alcohol. They may feel anxious or wish to 'block out' symptoms or side effects of medication. Or they may feel bored, lonely, have difficulties in sleeping or wish to boost self-confidence.

Alternatively people may start by misusing drugs or alcohol. Although this is not thought to cause mental health problems, it can trigger mental illness in people who have a predisposition.

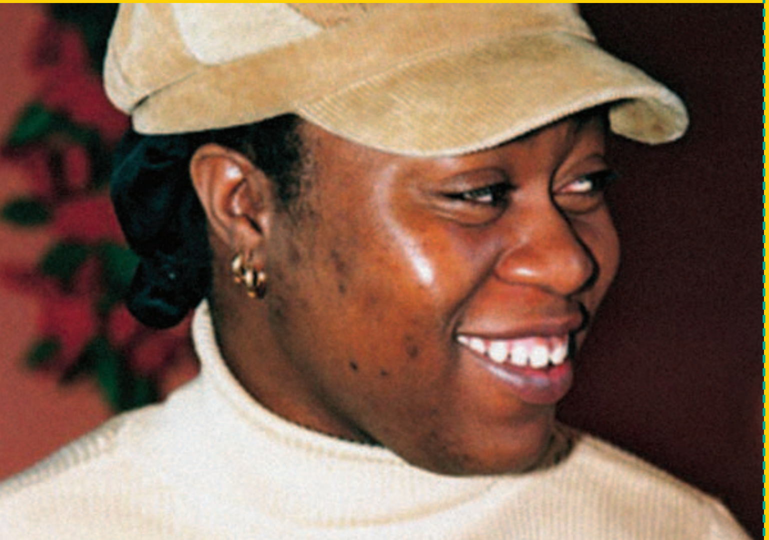

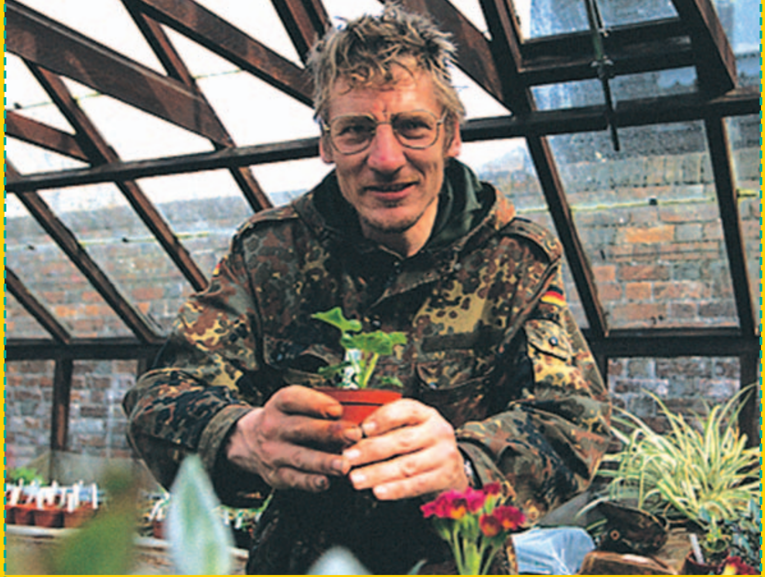

How many people are affected?

Whilst there is a need for further research, the evidence suggests that the numbers are high. A study in a London service showed that nearly a third of psychiatric patients were dependent on alcohol and 16% misused drugs.

Is it difficult to diagnose?

Yes, because the symptoms of each problem may be similar.

However, this is an area where family and friends can help by providing as much information as possible about the person. This can clarify both the factors that led to substance misuse and identify which is the primary problem.

What care is needed?

People will need a range of care from psychiatric services, psychological and social support services, and drug/alcohol agencies. It is often helpful to also have input from voluntary organisations.

Ideally, there would be co-ordination between all these services. In practice, staff in mental health are often not trained in addictions and vice versa. Hence, people can fall between services or have only part of their problem treated. Another factor in accessing care is the extent to which services require that patients should abstain from substance misuse before they are eligible for treatment for their mental health problem.

Currently there is a shortage of specific dual diagnosis services and a need for greater investment. If you are acting as an advocate for a friend or family member, you can ask whether the following resources are available:

- An experienced case manager who can co-ordinate a range of agencies
- Regular monitoring and outreach services so that the patient does not "slip through the net"'
- Education about the effects of alcohol/drug use
- Services that are sensitive to the needs of different cultures
- Help with coping skills such as recognising symptoms, dealing with the anxiety of withdrawal and preventing relapse
- Talking treatments such as cognitive-behavioural therapy
- Help for the patient in developing support systems in the community
- Support for you as a carer or friend

How can I support the care process?

Clarifying treatment options, waiting times and the proposed process is a good way of ensuring that you and your loved one feel informed and involved. Other areas for questioning might include: what the treatment goal is (eg total abstinence /maintenance/reduction of risk), the extent to which you can be involved, the likely effects of treatment, likely support needs, how you can spot the signs if treatment is not working, and what you should do in that instance.

In some cases, the person may request that friends and family are not informed or involved. This can be devastating and it may be particularly important for you to seek advice and support. It may be that you can show your care through background support rather than through specific involvement in treatment.

What are the effects of substance misuse?

Substances can be divided into broad groups with a range of effects:

Stimulants (uppers)

Include cocaine/crack, amphetamines and ecstasy. These generally stimulate and speed up body processes. They are risky for people with heart and blood pressure problems and can also cause or mimic mania, anxiety, depression or paranoid psychosis.

Depressants (downers)

Include opiates and opioids such as heroin, varieties of painkillers, tranquillisers and alcohol.These can cause loss of motivation and interest in surroundings, other people or oneself. Self-neglect, self-harm and even suicide can follow. It is easy to overdose on downers particularly where there is alcohol use.

Hallucinogens

Include cannabis, ecstasy, LSD and magic mushrooms. These cause intensified and mixed up sensations, delusions, hallucinations, impaired judgement and reasoning. Skunk, a noxious smelling product, is a stronger 'home grown' variety of cannabis that can cause hallucinations and paranoia. Hallucinogens are unpredictable and can trigger a latent mental illness.

Volatile Substances

Varieties of solvents such as glue, aerosols and lighter gas fuel. These can act as either stimulants or depressants. They can also cause people to behave in an uninhibited way and to experience hallucinations.

Can substance misuse make a mental health problem worse?

Yes, in a number of ways. These include making symptoms worse; provoking relapse and more frequent re-hospitalisation; causing social and behavioural problems; increasing the risk of suicide; adding physical health risks such as HIV, poor eating habits, liver damage; or complicating diagnosis and hence access to treatment.
