# Supplementary material for: A Controlled Trial of the Knowledge Impact of Tuberculosis Information Leaflets among Staff Supporting Substance Misusers: Pilot Study
Source: PLoS One. 2011 Jun 17;6(6):e20875. doi: 10.1371/journal.pone.0020875 (PMC3117836; doi:10.1371/journal.pone.0020875)
Supplement: Questionnaire S1 — National Knowledge Service TB : PART 1 Evaluation of information resource :TB & substance misuse: Guidance for substance misuse – key workers. 2008. Health Protection Agency. (PDF) [file pone.0020875.s003.pdf]

PART 1 Evaluation of information resource

TB & substance misuse: Guidance for substance misuse – key workers

---

Kindly answer the questions below BEFORE reading the leaflet.

---

1. Please indicate whether your answers are based on leaflet for :

Substance misuse & TB: Key worker ☐ Other ☐

2. Have you read any leaflet on TB before?

Yes ☐ No ☐ If yes – where ____

3. Have you attended any TB awareness / events on TB?

Yes ☐ No ☐ Not sure ☐ ____

4. Has anyone in your care known anyone, to have had TB?

Yes ☐ No ☐ If yes – how many approximately per year ____

5. How do you rate your knowledge of TB?

Very good ☐ Good ☐ Average ☐ Below average ☐ Poor ☐

Please note that the following is not a test. The answers will help us improve these resources for your benefit. All responses will be anonymous. (Kindly tick as many boxes as you think is appropriate)

---

6. Which form of TB is infectious?

Liver ☐ Lung ☐ Lymph Node ☐ Spine ☐ Brain ☐

7. Do you think TB is curable?

Don't know ☐ Yes ☐ No ☐

8. How long does TB treatment need to be given?

Don't know ☐ 6 – 9 days ☐ 6 – 9 weeks ☐ 6-9 months ☐ 6 – 9 years ☐

9. What are the symptoms of TB affecting the lungs?

Itchiness ☐
Persistent high temperature ☐
Unexpected bone fractures ☐
Heavy sweating at night ☐
General & unusual sense of tiredness ☐
Stomach cramping or bloating ☐
Loss of weight ☐
Cough – for a long time (2 -3 weeks or more) ☐
Coughing up blood ☐

10. What would you do if you suspected a client in your care has TB?

Do nothing
Refer to their GP or a Specialist healthcare team (if they exist)

11. What would you do if you suspected a client in your care is diagnosed with TB?

Client / relative will be given tablets, and will be responsible for taking then ☐
You or member of your staff will be asked to watch the clients take their tablets ☐
Your client's progress will be monitored more closely ☐
TB treatment will not interfere with any other medication they may be on ☐
My colleagues will be at higher risk of getting infected ☐
The client should not share house hold items such as bed linen, crockery and utensils ☐
Pay for their medication ☐

12. How can you help the medical services?

Transfer the client to another hostel ☐
Provide information about the client's background ☐
Support DOT ☐
Contact the Public Health lab ☐

Your professional background / organization:

Thank you for your co-operation, your participation is greatly appreciated and your efforts will help us improve the information resources we develop for you

Consent: I agree to take part in the above study and I understand that any information I provide will not be shared on an identifiable form and my participation is voluntary.
