# Supplementary material for: A Controlled Trial of the Knowledge Impact of Tuberculosis Information Leaflets among Staff Supporting Substance Misusers: Pilot Study
Source: PLoS One. 2011 Jun 17;6(6):e20875. doi: 10.1371/journal.pone.0020875 (PMC3117836; doi:10.1371/journal.pone.0020875)
Supplement: Questionnaire S2 — National Knowledge Service TB : PART 2 Evaluation of information resource : TB & substance misuse: Guidance for substance misuse - key workers. 2008. Health Protection Agency. (PDF) [file pone.0020875.s004.pdf]

PART 2 Evaluation of information resource

TB & substance misuse: Guidance for substance misuse – key workers

---

Please note that this is not a test – just something to help us develop resources for your benefit.

Please answer this questionnaire ***after*** reading the questionnaire: (*Kindly tick <u>as many boxes</u> as you think is appropriate)*

<u>1. Please indicate whether your answers are based on leaflet for :</u>

Substance misuse & TB: Key worker ☐ Other ☐

<u>2. Which form of TB is infectious?</u>

Liver ☐ Lung ☐ Lymph Node ☐ Spine ☐ Brain ☐

<u>3. Do you think TB is curable?</u>

Don't know ☐ Yes ☐ No ☐

<u>4. How long does TB treatment need to be given?</u>

Don't know ☐ 6 – 9 days ☐ 6 – 9 weeks ☐ 6-9 months ☐ 6 – 9 years ☐

<u>5. What are the symptoms of TB affecting the lungs?</u>

Itchiness ☐
Persistent high temperature ☐
Unexpected bone fractures ☐
Heavy sweating at night ☐
General & unusual sense of tiredness ☐
Stomach cramping or bloating ☐
Loss of weight ☐
Cough – for a long time (2 -3 weeks or more) ☐
Coughing up blood ☐

6. What would you do if you suspected a client in your care has TB?

Do nothing ☐
Refer to their GP or a Specialist healthcare team (if they exist) ☐

7. What would you do if you suspected a client in your care is diagnosed with TB?

Client / relative will be given tablets, and will be responsible for taking then ☐
You or member of your staff will be asked to watch the clients take their tablets ☐
Your client's progress will be monitored more closely ☐
TB treatment will not interfere with any other medication they may be on ☐
My colleagues will be at higher risk of getting infected ☐
The client should not share house hold items such as bed linen, crockery and utensils ☐
Pay for their medication ☐

12. How can you help the medical services?

Transfer the client to another hostel ☐
Provide information about the client's background ☐
Support DOT ☐
Contact the Public Health lab ☐

Your professional background / organization:

Thank you for your co-operation, your participation is greatly appreciated and your efforts will help us improve the information resources we develop for you

Consent: I agree to take part in the above study and I understand that any information I provide will not be shared on an identifiable form and my participation is voluntary.
